# Acute and chronic kidney complications in children with type 1 diabetes mellitus

**DOI:** 10.1007/s00467-022-05689-w

**Published:** 2022-07-27

**Authors:** Giulio Rivetti, Brenden E. Hursh, Emanuele Miraglia del Giudice, Pierluigi Marzuillo

**Affiliations:** 1 Department of Woman, Child and of General and Specialized Surgery, Università degli Studi della Campania “Luigi Vanvitelli”, Via Luigi De Crecchio 2, 80138 Naples, Italy; 2 Department of Pediatrics, Division of Endocrinology, British Columbia Children’s Hospital and University of British Columbia, 4480 Oak Street, Vancouver, BC V6H 3V4 Canada

**Keywords:** Acute kidney injury, Type 1 diabetes mellitus, Diabetic kidney disease, Chronic kidney disease, Children

## Abstract

Children with type 1 diabetes mellitus (T1DM) have an increased risk of developing kidney involvement. Part of the risk establishes at the beginning of T1DM. In fact, up to 65% of children during T1DM onset may experience an acute kidney injury (AKI) which predisposes to the development of a later chronic kidney disease (CKD). The other part of the risk establishes during the following course of T1DM and could be related to a poor glycemic control and the subsequent development of diabetic kidney disease. In this review, we discuss the acute and chronic effects of T1DM on the kidneys, and the implications of these events on the long-term prognosis of kidney function.

## Introduction

Children with type 1 diabetes mellitus (T1DM) can present with kidney involvement both in the acute setting presenting with acute kidney injury (AKI), as well as tubular damage and in the chronic setting presenting with diabetic kidney disease (DKD) [1–6].

It has been shown that during the onset of T1DM, an AKI—associated or not with tubular damage biomarkers—can occur in 43.8% of patients [1]. Moreover, separately evaluating patients with diabetic ketoacidosis (DKA), the prevalence of AKI significantly increases up to 65% [1, 2]. A further increase of prevalence of AKI up to 81% has been observed in cases of recurrent DKA episodes [7, 8]. Although both AKI and tubular damage are reversible, they have been associated with an increased risk of future chronic kidney disease (CKD) [2].

Furthermore, patients with T1DM may eventually develop DKD in 15–20% of cases, which in susceptible patients begins soon after the disease onset and may accelerate during adolescence [4].

For these reasons, kidney health for children with T1DM must not be neglected at each step of illness in order to avoid the development of CKD and a possible future progression towards kidney failure.

In this review, we discuss the acute and chronic effects of T1DM on the kidneys, and the implications of these events on the long-term prognosis of kidney function.

## Acute setting

According to the Kidney Disease/Improving Global Outcome (KDIGO), AKI can be defined by an increase of the serum creatinine or a reduction of the urine output [9].

Since diabetic patients usually present polyuria and polydipsia during the onset of the disease, the urine output criterion seems to be less reliable for the diagnosis of AKI. In fact, only 15% of patients with AKI at T1DM onset met the urinary output KDIGO criteria [1].

Nevertheless, clinicians should keep in mind that serum creatinine measurement in DKA is challenging, as high acetoacetate, glucose, and HbA1c levels may lead to a falsely elevated measured creatinine by interference with its measurement [10–13]. This effect is most pronounced with the Jaffe method at low creatinine concentrations, but is still observed with other enzymatic assay testing [10, 11].

### Pathophysiology of kidney damag﻿e

During the T1DM onset, the main pathophysiological mechanism is represented by hyperglycemia, which causes osmotic polyuria [1, 2]. The osmotic polyuria, in turn, leads to dehydration, hypovolemia, and kidney hypoperfusion, which causes tubular damage. This leads to an adaptive fall in glomerular filtration rate, due to the vasoconstriction which is an attempt to compensate the failure to reabsorb filtered solutes [14] with further deterioration of kidney perfusion [1, 2, 8]. A delayed therapeutic intervention may lead to the persistence of glomerular vasoconstriction, which may result in an acute tubular necrosis (described in about 30% of patients with T1DM onset [1]), thus shifting AKI from functional to intrinsic [15, 16]. For this reason, there has been a recent focus on the investigation of early tubular AKI biomarkers, in order to diagnose functional AKI in a timely manner, and to hopefully prevent the evolution to intrinsic AKI [3, 17].

When T1DM onset is complicated by DKA, acidosis could lead to further deterioration of tubular damage [1] with a further negative effect on kidney perfusion. Moreover, if impaired consciousness is present, the compensatory polydipsia disappears, which further deteriorates hydration status and kidney function.

Transient (< 48 h) AKI episodes are associated with better outcomes than persistent (2–7 days) ones, but still are not without consequences compared to no AKI [18, 19].

### Risk factors for AKI in children at T1DM onset and in those with known T1DM

#### AKI with DKA

AKI and DKA seem to be intriguingly interconnected. The more severe the DKA, the higher the probability of developing AKI [2, 7].

Lower bicarbonate [1, 2, 7, 20], higher heart rate (HR) [1, 2, 20], higher ketones [1], and higher chloride levels [21] (which are all factors related to DKA) [22–24] have been associated with AKI development at T1DM onset. Other factors associated with AKI in this setting are kidney length > 2 standard deviation scores (SDS), Ht ≥ 45% [1], male gender [7], higher corrected serum Na [1, 2, 20, 25], higher blood urea nitrogen, higher serum K^+^, and higher blood glucose levels [20]. These factors, however, do not present the same diagnostic value at all AKI stages. For example, ketones, HR > 2 SDS, Ht ≥ 45%, higher corrected serum Na levels, and kidney length > 2 SDS are associated with severe AKI but not with mild AKI [1].

Moreover, it has been shown that older age, recurrent DKA episodes, increased acidosis severity, increased time to anion gap normalization, and increased initial glucose are associated with a prolonged AKI recovery [7].

Patients with family history of T1DM have a significantly shorter duration of polyuria and polydipsia before T1DM diagnosis and lower prevalence of AKI when compared with those without T1DM family history [1]. This may reflect that their parents are aware of the signs and symptoms heralding T1DM [1]. This highlights that a prompt diagnosis might reduce the risk of developing AKI.

The evidence regarding the risks of developing AKI in patients with known T1DM is poor. Yang et al. found that longer duration of T1DM was an independent predictor of severe AKI in pediatric DKA with T1DM [8]. Moreover, we can assume that the patients with the greatest risk of developing AKI are those with recurrent DKA who, in turn, present a prolonged AKI recovery [7], itself associated with increased risk of CKD [26]. Therefore, in recurrent DKA, the risk of AKI could be higher because of (i) the higher T1DM duration and (ii) possible previous AKI episodes which could reduce the “functional reserve of the kidneys.”

#### AKI without DKA

The current literature principally focuses on the incidence of AKI during DKA, since during T1DM onset the development of AKI is deeply related to DKA severity.

Nevertheless, as about one-fifth of patients with T1DM onset without DKA develops AKI [1], we want to highlight that it is extremely important to pay heed to kidney function and focus on a proper rehydration in this group of patients, who are generally considered at a lower risk of severe presentation of diabetes and of severe complications of initial diabetes therapy.

The DiAKIdney is the only study in which this population is described, identifying serum chloride level as the most important risk factor for AKI in patients without DKA [1].

### Prevalence and risk factors for tubular damage in children at T1DM onset and in those with known T1DM

No universal definition of tubular damage has been provided. The fractional excretion of sodium is an important and easily available marker to help identify the kind of kidney involvement. On the other side, the tubular reabsorption of phosphate can be supportive in identifying a tubular involvement.

Piani et. al reported that DKA is characterized by markers of reversible tubular injury and that the degree of injury is associated with elevated copeptin and serum uric acid (SUA) levels in children with T1DM [3]. In fact, during the onset of T1DM, high levels of copeptin and SUA are associated with the presence of tubular injury markers, such as neutrophil gelatin–associated lipocalin (NGAL), kidney injury molecule 1 (KIM-1), chitinase 3-like 1 (YKL-40), interleukin 18 (IL-18), and monocyte chemoattractant protein-1 (MCP-1) [3].

Aminoaciduria can also be considered a marker of tubular dysfunction, since an increased excretion of amino acids has been reported during DKA [27–29]. Recently, Melena et al. demonstrated that DKA is associated with a profound aminoaciduria, suggestive of proximal tubular dysfunction, similarly to Fanconi syndrome [17]. In fact, it has been hypothesized that a proximal tubular injury and the consequent aminoaciduria may serve as a marker of early functional and structural damage in the kidney and may represent an early indicator of kidney disease development and progression in T1DM. In particular, during DKA, it was found that the concentration of urine histidine, threonine, tryptophan, and leucine per creatinine are higher at 0–8 h and then significantly decrease over 3 months. Moreover, in patients with severe DKA, there is significantly elevated urinary excretion of leucine compared to those who experience mild DKA, who in turn have a lower excretion of tryptophan compared to those who have a moderate DKA [17].

However, a general tubular damage (either associated to subclinical or overt AKI) can be observed in up to 73.5% of patients at T1DM onset [1]. Indeed, high glucose states such as DKA have been shown to induce a proximal tubular degeneration [20]. Moreover, around 30% of patients with T1DM onset present with increased tubular biomarkers without a real kidney dysfunction (subclinical AKI) [30, 31] and, on the other hand, almost 12% of patients may have a functional loss that occurs in the absence of detectable kidney damage, based on biomarkers (hemodynamic AKI) [1, 31].

Many risk factors have been identified for each combination between kidney and tubular involvement. The highest serum creatinine at T1DM onset/basal creatinine (HC/BC) ratio indicating a more severe AKI was significantly associated with acute tubular necrosis [1]. Also, kidney length > 2 SDS was significantly associated with hemodynamic AKI, while lower serum phosphorus levels and higher HC/BC ratio were significantly associated with subclinical AKI [1].

Lastly, although there is no study dealing with the incidence of tubular damage in patients with known diabetes, we could assume that patients with recurrent DKA are at higher risk of developing tubular damage, such as already hypothesized for AKI risk.

### Clinical management

The clinical management of DKA is fully described in the ISPAD 2018 guidelines [32]. Fluid replacement is of paramount importance, especially as we consider kidney health, and it should begin before starting insulin therapy.

As pointed out before, AKI can occur during the acute onset of T1DM, due to acidosis, dehydration, and hypovolemia. Considering that an episode of AKI is independently associated with an increased risk of CKD [1] and hypertension [33], and that in turn hypertension is an important risk factor to develop DKD (that eventually leads to CKD), we can realize how important it is to quickly initiate a safe rehydration therapy during T1DM onset. Hence, we suggest that a “therapeutic compromise” between a too slow fluid replacement therapy (that could lead to an AKI and all of the further consequences described) and a too rapid replacement (that on the other hand could lead to a cerebral edema) should be found. Laskin et al. suggested to give fluids to patients with AKI secondary to volume depletion while quickly shifting to more restrictive strategies in those who do not respond to volume and have decreasing urinary output [34]. The data deriving from the study of Kuppermann et al., however, are reassuring about the risk of neurologic outcomes in patients with DKA [35]. In fact, neither the rate of administration nor the sodium content of intravenous fluids significantly influenced neurological outcomes in children with DKA [35]. Future expert panels should identify the best treatment modalities, taking into account the kidney health of patients with T1DM onset.

## Chronic setting

In the past, chronic kidney involvement in patients with T1DM was defined as diabetic nephropathy. However, in 2020, the KDIGO guidelines suggested avoiding the term diabetic nephropathy because there is still no consensus definition [36]. For this reason, and because the term diabetic nephropathy is technically a histopathological diagnosis [37], in this manuscript we use the term DKD which indicates the clinical syndrome related to a chronic kidney involvement in patients with T1DM [6]. Therefore, DKD can be considered a clinical manifestation of the histopathological anomalies of diabetic nephropathy and the previous definitions of diabetic nephropathy from a practical point of view are overlapping with the term DKD [4, 5].

Many authors proposed their own definition of DKD, and almost all of them agreed with the ISPAD 2018 guidelines [5], in which the beginning of DKD is associated with the development of microalbuminuria (albumin excretion rate between 30 and 300 mg/24 h or 20 and 200 μg/min in a 24-h or timed urine collection). For example, in 2008, Bogdanovic stated that a clinically detectable DKD begins with the development of microalbuminuria [4]. This definition was lately confirmed by Parkins et al. suggesting that DKD can be defined by the development of microalbuminuria or by loss in GFR in patients affected by T1DM [38]. Particular attention should be paid to this latter criterion for the definition of DKD. In fact, a substantial proportion of patients with T1DM have a kidney function loss without an overt proteinuria or even with normo-albuminuria. This particular form of DKD is called nonproteinuric diabetic kidney disease and is defined by an eGFR < 60 mL/min/1.73 m^2^ and a urine albumin to creatinine ratio (Ua:CR) ≤ 300 mg/g creatinine [39].

We can finally summarize all these findings as we attempt to give a universal definition of DKD, which can be considered as a microvascular complication of the diabetes characterized by the development of microalbuminuria/proteinuria or by the reduction of eGFR in patients with T1DM.

### Pathophysiology of kidney damage

DKD is a dynamic process that can affect the kidney function and morphology over the years, and is sustained by a continuous exposure of the kidneys to high blood glucose levels [6, 40]. In fact, hyperglycemia causes an abnormal homeostasis in blood flow and a vascular permeability in the glomerulus. The increased blood flow and intracapillary pressure eventually leads to a decreased nitric oxide production on the efferent side of the glomerular capillaries, causing an increased sensitivity to angiotensin II with profibrotic effects. At the beginning, the increased permeability can be reversible, but under the continuous triggering effect of hyperglycemia, the lesions become irreversible [41]. DKD determines changes in the kidney structure over years and is schematically divided in 5 stages.

1. Hyperfiltration: with the onset of diabetes hyperglycemia usually determines kidney hemodynamic changes that end up with the constriction of the efferent arteriole and a glomerular hypertension that eventually determine a glomerular hypertrophy. In fact, during this first phase, there is an increased kidney size and increase of the eGFR by 20–40% [42]. Microalbuminuria can be present during this phase, but it is usually reversible with insulin treatment and there is no evidence of histological lesions in glomeruli or vascular structure [4].
2. Silent: during this phase, there is a thickening of the glomerular basal membrane and a mesangial matrix expansion caused by the production of reactive oxygen species [42] that is typically related to the high glucose exposure of these tissues. In fact, microalbuminuria can be present in this stage, but only during the periods of poor metabolic control or with exercise.
3. Incipient: about 7–10 years after the diagnosis, microalbuminuria appears in 1/3 of the patients. Microalbuminuria is considered as the very first clinical sign of DKD and is often associated with established significant glomerular damage: during this phase there is an increase in blood pressure (BP) (about 3 mmHg/year), albeit still within the conventional age-corrected normal range. In fact, in adolescents, microalbuminuria can be preceded by an increase of the nocturnal systolic BP [43].
4. Overt: this stage is characterized by an overt proteinuria (> 0.5 g/24 h), a steady rise of BP, an increased albumin excretion rate and the decline of glomerular filtration rate (GFR) by about 10 ml/min year. This stage occurs 10 to 15 years after T1DM onset and is highly predictive of subsequent progress to kidney failure, if left untreated [41].
5. Kidney failure: the final stage is characterized by uremia and can occur in up to 40% of T1DM patients usually 10 years after the appearance of proteinuria [4].

With a worse patient compliance to insulin therapy and higher blood glucose levels, the terminal phase (and the need of a kidney function replacement therapy) is reached more quickly.

### Prevalence and risk factors for diabetic kidney disease

Approximately 20 to 30% of people with T1DM have microalbuminuria (and consequently DKD) after a mean diabetes duration of 15 years and the overall incidence of kidney failure is reported to be 4 to 17% at 20 to 30 years from T1DM diagnosis [41].

**Fig. 1:**
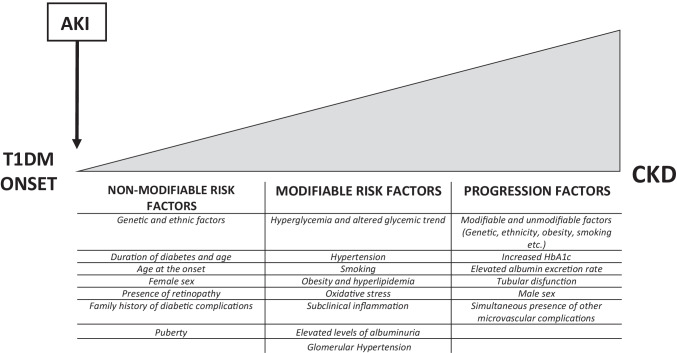
Risk factors for development of diabetic kidney disease. The development of diabetic kidney disease is a dynamic process and is the result of cumulative kidney insults. Classically, non-modifiable and modifiable risk factors and progression factors can be identified. In addition, recent evidence indicates the importance of the potential kidney damage as consequence of the severity of T1DM onset

Many risk factors are related to the development of DKD and they can be divided in non-modifiable and modifiable risk factors as well as in factors predisposing to progression to CKD (Fig. 1) [4, 44]. The long-term glycemic control is the most important factor for the development and severity of complications in T1DM. A causal relationship between chronic hyperglycemia and diabetic microvascular complications has been demonstrated [4].

The level of albuminuria instead has been shown to predict the progression to CKD stage 5 [45, 46], and it is associated with an increased risk of macrovascular disease [47].

Translating the evidence about the relationship between AKI and CKD, in our opinion AKI itself could be considered a risk factor for DKD. The results of the study by Huang et al. [48] reinforce our hypothesis. They, indeed, demonstrated how each episode of AKI during DKA can be associated with a hazard ratio of 1.56 for development of microalbuminuria that can increase by more than fivefold if four or more episodes of AKI occur [48].

**Fig. 2:**
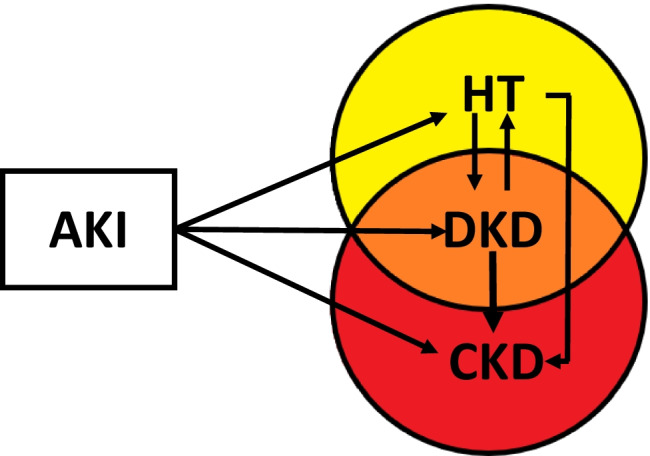
The relationship between AKI, hypertension, diabetic kidney disease, and chronic kidney disease. AKI, while it can be totally reversible, may also lead to subclinical damage which predisposes to hypertension (HT), diabetic kidney disease (DKD), and chronic kidney disease (CKD). HT itself could determine progression toward DKD or CKD

In addition to the AKI-related CKD risk, it has been proven that an AKI is independently associated with a 22% increase of the odds of developing hypertension which is in turn related to DKD [33]. DKD, in turn, eventually leads to CKD (Fig. 2). In fact, high BP and alterations in circadian rhythm have been associated with the risk of developing nephropathy and retinopathy in youth with T1DM [49].

### Clinical management

Screening for DKD should begin at 11 years with 2–5 years diabetes duration [5]. A regular annual follow-up is important to identify a rapid or slow progression to microalbuminuria, as well as cases of regression to normo-albuminuria.

Furthermore, longitudinal follow-up of albumin excretion is also important to identify patients with progressive small increases of the urinary albumin excretion within the normal range, which might be a prelude to the development of microalbuminuria.

Patients with DKD eventually develop hypertension, in fact 10 years after the onset of the disease an increase of BP can occur [4]. The relationship between hypertension and DKD can be explained by the retention of concentrated sodium and subsidiary blood vessel resistance [50].

Various pediatric diabetes clinical practice guidelines suggest measuring BP at least annually or twice-yearly for children with T1DM [51]. As indicated in the current pediatric hypertension guidelines [52], oscillometric devices may be used for BP screening in children and adolescents. If elevated BP is reported by the oscillometric readings, confirmatory measurements should be obtained by auscultation. An appropriately sized cuff should be used for accurate BP measurement and if the initial BP is elevated (≥ 90^th^ percentile), two additional oscillometric or auscultatory BP measurements should be performed at the same visit and averaged [52]. To confirm the diagnosis of hypertension according to casual BP measurements, a subsequent ambulatory BP monitoring (ABPM) should be performed [52].

Considering that even a mild episode of AKI stage 1 can double the risk of CKD [53], an anticipation of the need for screening for DKD and a closer follow-up for this cohort of patients, especially in the case of recurrent DKA, could be required. In fact, according to the KDIGO guidelines, after an AKI episode a follow-up is necessary in order to detect the development of proteinuria and hypertension which herald CKD [9].

Urine albumin–creatinine ratio (Ua:CR) measured after AKI is a strong and potentially modifiable risk factor for more rapid loss of kidney function [54]. Stoumpos et al. reported that even among patients who had severe AKI requiring dialysis, those who had a post-AKI eGFR level greater than 60 mL/min/1.73 m^2^ had a low risk of accelerated loss of kidney function [55]. However, Hsu et al. showed that proteinuria is more significantly associated with a subsequent loss of kidney function than post-AKI eGFR level [54]. This, in our opinion, underlines the importance of an adequate proteinuria monitoring.

It has been recommended that patients with AKI should be monitored through the evaluation of:

- BP and Ua:CR, (on the first urine of the morning taken on rising) 12 months after AKI.
- Annual BP and Ua:CR for life.
- Serum creatinine if previous measurement elevated or if proteinuria or raised BP develops [14].

The clinical management of DKD is fully described in the ISPAD 2018 guidelines [5]. In summary, management is focused on achieving excellent glycemic management with HbA1c 7% or less [4]. Lifestyle modifications in the form of weight loss, dietary changes, and increased physical activity are not only useful for glycemic management, but they also aid in preventing the development of hypertension and treating existing hypertension. When hypertension is confirmed, pharmacologic treatment should be considered in addition to lifestyle modification.

Moreover, antiproteinuric drugs represent a pivotal treatment in cases of microalbuminuria, and renin–angiotensin–aldosterone system (RAAS) inhibitors are considered the mainstay of treatment for DKD. In fact, pharmacological renoprotective treatment with angiotensin-converting enzyme inhibitors (ACE-I) is indicated for all patients with persistent microalbuminuria regardless of BP measurements [56–58].

The principal mechanism of kidney protection by RAAS inhibitors is to reduce intraglomerular pressure and glomerular hyperfiltration [59, 60], and in addition to ameliorate angiotensin II-induced oxidative stress, inflammation, and fibrosis [61].

ACE-I are recommended for use in children and adolescents with hypertension and microalbuminuria [5]. Therapy with ACE-I is known to reduce the progression to overt nephropathy by 62% and increase the regression to normo-albuminuria three-fold compared with placebo [58]. Many studies have also confirmed that treatment with ACE-I may lead to the reduction [62, 63] or normalization [64, 65] of microalbuminuria and the preservation of normal GFR [66].

If this drug is not tolerated (e.g., due to cough), an angiotensin receptor blocker (ARB) can be used; indeed, this latter class is considered to have similar effects on lowering BP and decreasing albuminuria [67].

Many studies completed in hypertensive children showed that ACE-I (such as enalapril [68], lisinopril [69], and ramipril [70]) and ARBs (such as irbesartan [71], telmisartan [72], valsartan [73], candesartan [74], and losartan [75]) had few adverse effects.

Since RAAS inhibition has been shown to improve the prognosis in patients with DKD [76], we agree that persistent microalbuminuria should be treated regardless of BP measurements, using the lowest effective dose for the treatment progressively increased up to a maximum safe dose until the regression of microalbuminuria is achieved. This latter was defined by a reduction of 50% or more in the albumin excretion rate from one 2-year period to the next [77].

The combination of ACE-I and ARBs has been shown to have additional renal protective effects in albuminuric adults with diabetes [78], even if it is not recommended in pediatric DKD, partly because of the increased risk of acute-on-chronic kidney impairment and hyperkalaemia [79].

Lastly, we can summarize the three main targets of management in the chronic setting:

- To obtain the best glycemic control maintaining HbA1c levels at 7% or less
- To maintain BP in the normal range, defined by systolic and diastolic BP values < 90th percentile (on the basis of age, sex, and height percentiles)
- To detect microalbuminuria early on and achieve regression of microalbuminuria.

## Conclusions: kidney health in T1DM patients, an integrated overview

Part of the future risk of developing CKD in T1DM is established at the onset of diabetes. Indeed, up to 65% of the patients at T1DM onset can develop AKI, which in turn is associated with an increased risk of CKD. The more severe the AKI episode, the higher the associated risk of developing CKD and kidney failure [53].

As a preventive measure, a higher parental awareness to the red flags of T1DM should be provided by Pediatricians in order to facilitate an early diagnosis of T1DM reducing the risk of AKI at T1DM onset and then of later CKD [1].

In addition to the “first hit” to kidneys at T1DM onset, during the years of the illness additional hits can further deteriorate the kidney function such as recurrent DKA with recurrent concomitant AKI. AKI, however, can also develop in non-diabetes-related conditions such as acute gastroenteritis or community acquired pneumonia [80, 81]. Therefore, T1DM patients should be carefully informed about the importance of dehydration prevention by adequate hydration in case of the common acute illnesses of childhood.

Moreover, poor glycemic control predisposes to DKD development with subsequent risk of CKD, indicating the importance of an adequate compliance to T1DM treatment.

Finally, in patients with T1DM regular follow-up visits are important to identify the possible onset of microalbuminuria or hypertension to start a timely and adequate treatment. These conditions, in fact, can facilitate both onset and progression of CKD.

### Key summary points

1. At T1DM onset, AKI occurs in 1/5 of patients without DKA and 2/3 of patients with DKA.
2. With more severe onset of T1DM, there is higher risk of AKI and subsequent increased risk of CKD.
3. DKD is a microvascular chronic complication of T1DM. It can occur in almost 1/5 of patients.
4. Poor glycemic control and previous AKI episodes increase the risk of developing DKD.
5. Regular T1DM follow-up visits are important to identify the possible onset of microalbuminuria or hypertension. Timely and adequate treatment with renin–angiotensin–aldosterone system inhibitors should be considered.

### Multiple Choice Questions (answers can be found following the reference list)

1. Acute kidney injury in children at T1DM onset…
  - … could manifest in about 2/3 of children with DKA
  - … is extremely rare
  - … is usually not reversible
  - … could manifest in about of 2/3 of children without DKA
2. In AKI pathophysiology for patients at the onset of T1DM, all of the following factors are involved with the exception of
  - osmotic polyuria
  - delayed T1DM diagnosis
  - acidosis
  - older age at T1DM onset
3. The presence of diabetic kidney disease may be indicated by all the following parameters with the exception of:
  - urine albumin to creatinine ratio > 30 mg/g creatinine
  - eGFR < 60 mL/min/1.73 m.^2^
  - urine albumin to creatinine ratio > 300 mg/g creatinine
  - glycosuria
4. The first-choice pharmacological treatment for hypertension in children with T1DM is
  - angiotensin-converting enzyme inhibitors
  - calcium channel blockers
  - diuretics
  - beta-blockers
5. The risk of developing DKD increases in case of:
  - poor glycemic control
  - previous AKI episode
  - untreated hypertension
  - all of the above

## Data Availability

Not applicable.
